# Exercise training increases GAD65 expression, restores the depressed GABA_A_ receptor function within the PVN and reduces sympathetic modulation in hypertension

**DOI:** 10.14814/phy2.14107

**Published:** 2019-07-01

**Authors:** Nilson C. Ferreira‐Junior, Adriana Ruggeri, Sebastião D. Silva, Thais T. Zampieri, Alexandre Ceroni, Lisete C. Michelini

**Affiliations:** ^1^ Department of Physiology & Biophysics Institute of Biomedical Sciences University of Sao Paulo Sao Paulo SP Brazil

**Keywords:** Autonomic nervous system, bicuculline, exercise, GABA_A_ receptors, glutamic acid decarboxylase, hypothalamus, spontaneous hypertension

## Abstract

GABAergic inhibitory input within the paraventricular hypothalamic nucleus (PVN) plays a key role in restraining sympathetic outflow. Although experimental evidence has shown depressed GABA_A_ receptor function plus sympathoexcitation in hypertension and augmented GABA levels with reduced sympathetic activity after exercise training (T), the mechanisms underlying T‐induced effects remain unclear. Here we investigated in T and sedentary (S) SHR and WKY: (1) time‐course changes of hemodynamic parameters and PVN glutamic acid decarboxylase (GAD) isoforms’ expression; (2) arterial pressure (AP) and heart rate (HR) responses, sympathetic/parasympathetic modulation of heart and vessels and baroreflex sensitivity to GABA_A_ receptor blockade within the PVN. SHR‐S versus WKY‐S exhibited higher AP and HR, increased sympathetic reduced parasympathetic modulation, smaller baroreflex sensitivity, and reduced PVN GAD65 immunoreactivity. SHR‐T and WKY‐T showed prompt maintained increase (2–8 weeks) in GAD65 expression (responsible for GABA vesicular pool synthesis), which occurred simultaneously with HR reduction in SHR‐T and preceded MAP fall in SHR‐T and resting bradycardia in WKY‐T. There was no change in GAD67 expression (mainly involved with GABA metabolic pool). Resting HR in both groups and basal MAP in SHR were negatively correlated with PVN GAD65 expression. Normalized baroreflex sensitivity and autonomic control observed only in SHR‐T were due to recovery of GABA_A_ receptor function into the PVN since bicuculline administration abolished these effects. Data indicated that training augments in both groups the expression/activity of GABAergic neurotransmission within presympathetic PVN neurons and restores GABA_A_ receptors′ function specifically in the SHR, therefore strengthening GABAergic modulation of sympathetic outflow in hypertension.

## Introduction

Despite intense scientific research and advances in the diagnosis, control and prevention of cardiovascular diseases, hypertension is a major health problem worldwide affecting about 20% of the adult and over 50% of the elder population. Arterial hypertension leads to myocardial infarction, stroke, renal failure, and death if not detected early and treated appropriately (James 2014). In between the mechanisms conditioning hypertension, recent studies have renewed the importance of the sympathetic nervous system since hypertension is accompanied by sympathoexcitation and adrenergic overactivity has been reported as an independent predictor of mortality in several diseases (Grassi et al. 2015a,b).

The presympathetic neurons located in the hypothalamic paraventricular nucleus (PVN) play a prominent role in the generation/modulation of sympathetic vasomotor tone specifically in disease states involving sympathetic overactivity (Coote et al. 1998; Allen 2002; Dampney et al. 2005). They project to both sympathetic premotor neurons in the rostroventrolateral medulla (RVLM) and sympathetic preganglionic neurons located in the intermediolateral cell column of the spinal cord, being a major source of excitatory drive to sympathetic outflow (Ciriello et al. 1985; Dampney et al. 1987; Hardy 2001). The PVN presympathetic neurons receive both tonic excitatory (glutamatergic) and inhibitory (GABAergic) inputs, the balance of which determines the excitability of the PVN presympathetic neurons (Li and Pan 2007; Dampney et al. 2018). In spontaneously hypertensive rats (SHR) Li & Pan (2006) showed that the increased sympathetic outflow was accompanied by reduced GABAergic inhibitory postsynaptic currents in PVN neurons projecting to the RVLM. By administering agonists and antagonists of GABA_A_ and GABA_B_ receptors into the PVN they also showed in SHR an important attenuation of GABA_A_ receptor function (loss of GABAergic neurons, reduced receptors’ number) accompanied by enhanced presynaptic GABA_B_ activity, which regulates both glutamate (net increase) and GABA (marked reduction) release (Li and Pan 2006; Li et al. 2008). Since GABAergic neurotransmission accounts for 60% of total synapses within the PVN (Decavel and Van den Pol 1990), the imbalance between GABAergic (reduced inhibition) and glutamatergic synaptic inputs shall lead to hyperactivity of PVN presympathetic neurons and heightened sympathetic vasomotor tonus in hypertension (Dampney et al. 2018).

Accumulating experimental evidence has shown that aerobic exercise training, a nonpharmacological tool, reduces the sympathetic hyperactivity in different models of hypertension (Ceroni et al. 2009; Rossi et al. 2013; Jia et al. 2014; Masson et al. 2014) as in human beings (Cornelissen and Smart 2013; James 2014; Pescatello et al. 2015). Reduction of both sympathetic outflow and blood pressure have been attributed to training‐induced changes in neuronal circuitry involved in the genesis/modulation of autonomic circulatory control. Indeed, aerobic training downregulates brain renin‐angiotensin system, reduces angiotensin II and reactive oxygen species availability, increases antioxidant capacity, deactivates microglia and reduces the proinflammatory profile in autonomic brain areas as the PVN and RVLM (Felix and Michelini 2007; Pan et al. 2007; Agarwal et al. 2011; Masson et al. 2014; Chaar et al. 2015). It has been shown that changes in GABAergic neurotransmission also contributes to the improvement of cardiovascular control in trained SHR since exercise increased the expression of glutamic acid decarboxylase (GAD, the rate‐limiting enzyme for GABA synthesis), augmented GABA levels thus blunting the GABAergic deficit within the caudal hypothalamus and attenuating both renal sympathetic nerve hyperactivity and hypertension (Kramer et al. 2001; Little et al. 2001). Experimental evidence also showed that exercise training reduced GABAergic neurotransmission within the PVN (Rossi et al. 2013; Jia et al. 2014). However, there is no information on time‐course changes of PVN GABAergic modulation in trained rats and whether these effects are or are not temporarily correlated with cardiovascular responses. Also it is not known whether exercise training is able to rescue the depressed GABA_A_ function in hypertensive individuals.

Considering that PVN GABA availability and GABA_A_ receptor function of the SHR are markedly attenuated in the established phase of hypertension (Li and Pan 2006; Li et al. 2008), it is our hypothesis that training restores the GABAergic modulation of sympathetic activity by augmenting GABA availability and improving the activity of the ionotropic GABA_A_ receptor within the PVN. Since the two GAD isoforms may have different functional roles in GABAergic neurons (Kaufman et al. 1991; Soghomonian and Martin 1998) it is possible that training differentially affects GAD 65 and GAD67 expression. Therefore, in the present study we analyzed simultaneously in conscious adult SHR and age‐matched controls the temporal effects of aerobic training on cardiovascular parameters and gene and protein expression of both GAD isoforms. In addition, by recording blood pressure, heart rate and autonomic responses before and after bicuculline administration into the PVN of sedentary and trained rats, we evaluated the effects of exercise training on GABA_A_ receptor function.

## Materials and Methods

### Ethical approval

All experimental procedures were carried out in compliance with the Ethical Principles in Animal Research of our National Council for Control of Animal Experimentation (CONCEA), in accordance with the Guidelines for Research in Animals and Human Beings of the American Physiological Society. The protocols were reviewed and approved by the Institutional Animal Care and Use Committee (CEUA #142) of the University of Sao Paulo.

### Animals and experimental protocols

Two‐month‐old male Spontaneously Hypertensive Rats (SHR) and Wistar‐Kyoto (WKY) weighing 200–250 g at the beginning of experiments were used. Rats were obtained from the Animal Breeding Facility of the University of São Paulo and housed in the Animal Facility of the Department of Physiology & Biophysics, Biomedical Sciences Institute, under controlled temperature/humidity, 12 h/12 h light‐dark cycle (lights on at 6 am), with free access to water and standard laboratory chow. To evaluate the sequential effects of aerobic training on tonic inhibitory inputs in PVN neurons simultaneously with the cardiovascular responses and to validate their interaction, SHR and WKY were submitted to two experimental protocols: *Protocol I:* Time series analysis (1 up to 8 weeks) of hemodynamic parameters and GABAergic activity (GAD isoforms expression) within the PVN induced by aerobic exercise training or sedentary protocol; *Protocol II*: Analysis of cardiovascular responses induced by the blockade of GABA_A_ receptors (bicuculline) within the PVN in SHR and WKY trained for 4 weeks or kept sedentary (proof‐of‐concept to validate changes in PVN GABAergic tonus).

### Maximal exercise tests, aerobic exercise training, and sedentary protocols

**Figure 1:**
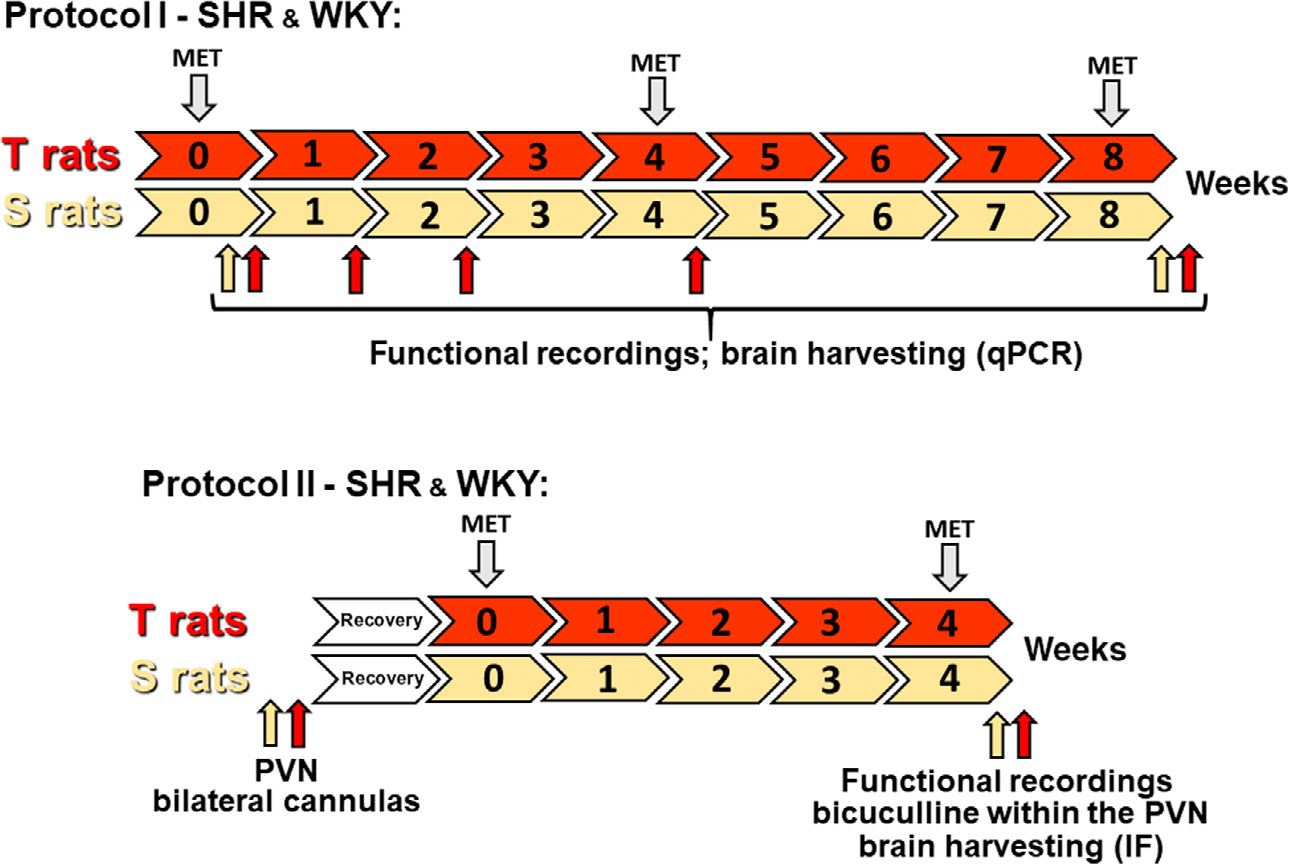
Timeline of the experimental design used in protocols I and II after a 2 weeks adaptation period to the treadmill. SHR and WKY rats were submitted to training (T) or sedentary (S) protocols for 8 (Protocol I) or 4 weeks (Protocol II). Maximal exercise tests (MET) to allocate rats with similar exercise capacity to T and S groups and establish the training intensity and to calculate the performance gain were performed at the beginning and end of protocols, respectively. In protocol I another MET was performed at the fourth experimental week for adjustment of training intensity in T rats. Functional recordings followed by brain harvesting were made at weeks 0, 1, 2, 4, and 8 (red and yellow arrows). Rats allocated to Protocol II were previously submitted to bilateral PVN cannulation, followed by 1‐week recovery before starting T and S protocols. Functional recordings, bicuculline administration and brain harvesting (red and yellow arrows) were performed at the end of experiments.

Rats used in *Protocol I* were preselected for their ability to walk/run on a treadmill (KT‐300, Inbramed, Porto Alegre, Brazil, 0.3 up to 0.9 km/h daily sessions, 10 min/day) during a 2 weeks adaptation period. As described previously (Buttler et al. 2017; Santos et al. 2018) only active rats were included in the experiments. Rats were then subjected to progressive maximal exercise tests (MET) until exhaustion in order to allocate rats with identical aerobic capacities to training and sedentary groups and to set the intensity of exercise training (Cavalleri et al. 2011). In a previous study MET was confirmed as a valuable index to quantify the training effect (Buttler et al. 2014). As depicted in Figure 1, SHR and WKY were submitted to low‐to‐moderate aerobic training [T = 50%–60% of maximal exercise capacity, 0% inclination, performed 1 h/day, 5 days/week, for 8 (*Protocol I*) or 4 weeks (*Protocol II*)]. Sedentary controls (S), housed in the same room, were handled 5 days/week and underwent a short exercise session once a week (0.3–0.9 km/h, 0% inclination for 10 min) to ensure similar stress conditions in both groups. METs were repeated at the fourth week for comparison of T and S gain (*Protocol II*) and adjustment of training intensity (*Protocol I*) and at the 8th week to compare the effectiveness of T and S during the 8 experimental weeks (*Protocol I*).

### Stereotaxic surgery and drug injection procedure

Rats used in *Protocol II* were submitted to chronic cannulation of the PVN before T or S protocols (Fig. 1). Rats were anesthetized (ketamine 100 mg/kg + xylazine 20 mg/kg *i.p.,* Sespo, Paulinia, SP, Brazil) and placed in stereotaxic apparatus (David Kopf, Tujunga CA). After local anesthesia (2% lidocaine chloridrate + 0.04% phenylephrine chloridrate, White, Rio de Janeiro, RJ, Brazil) and asepsis, the skull was exposed, small holes were opened bilaterally for implantation of the guide cannulas (Buttler et al. 2017) directed to the ventromedial nucleus of the medial PVN or to the posterior PVN nucleus according to the following coordinates: 1.8 mm or 2.2 mm caudal to the Bregma, 0.8 mm or 1.0 mm right and left of the medial suture and 7.2 or 7.0 mm ventral to the skull surface, respectively (Paxinos and Watson 1998). One metal screw fixed to the skull + the guide cannulas were cemented with fast polymerizing methacrylate. During surgery, the level of consciousness was frequently assessed by the degree of antinociception. Rats were then treated with a nonsteroidal anti‐inflammatory (flunixin meglumine, 5 mg/kg _*S.C*._., Banamine^®^, Schering‐Plough, Cotia, São Paulo, Brazil) and a poly‐antibiotic preparation of streptomycins and penicillins _*I.M*._ (Pentabiotico^®^, Fort Dodge, Campinas, Sao Paulo, Brazil) for analgesia and postoperative infection prevention, respectively. PVN cannulation was performed after 1‐week adaptation period to the treadmill. After a 7 days period for surgery recovery rats were submitted to another 1‐week treadmill adaptation and then to T or S protocols for 4 weeks.

PVN microinjections were made in conscious unrestrained rats. Microinjection needle (33G, 1 mm longer than guide cannula) was connected through a PE‐10 tubing to a 1‐*μ*L syringe (7002‐H, Hamilton Co., Reno, NV) and carefully inserted into the guide cannula. Bicuculline (50 pmol/100 nL, Sigma‐Aldrich, St. Louis, MO) was injected over a 30‐s period. The needle was removed and inserted into the contralateral guide cannula for bilateral microinjection into the PVN. The exact location of areas microinjected was confirmed at the end of experiments by Evans Blue administration (2%/100 nL).

### Chronic arterial catheterization and functional measurements

After the last training session at experimental weeks 0, 1, 2, 4, and 8 (*Protocol I*) and 4 (*Protocol II,* Fig. 1), rats recovered from 20–30 min and were anesthetized with ketamine and xylazine and placed on a heated surgical table (37°C). A chronic catheter (5 cm of 0.28:0.61connected to 15 cm of 0.50:1.50 ID;OD, Tygon Tubing, Critchley, Australia) was inserted into the abdominal aorta through the femoral artery, as described before (Ceroni et al. 2009). The external tubing was tunneled subcutaneously and exteriorized on the animal's dorsum, where it was fixed with a suture. Flunixin meglumine was applied for postoperative analgesia. Rats were placed in their home cages and rested for 24 h. Pulsatile arterial pressure (AP) and heart rate (HR) were recorded in the following day, at least 25–26 h after the last bout of exercise. The arterial cannula was connected to the recording system [transducer (CDX III Model, Cobe Labs, Lakewood, CO) + amplifier (ML224 Quad Bridge Amp, ADInstruments, New South Wales, Australia) + digital acquisition system (PowerLab, ADInstruments, New South Wales, Australia)], and 15–20 min were allowed for the cessation of the exploratory activity. Resting AP and HR were then continuously recorded during 30–40 min (computer, 2000 Hz of sampling frequency, LabChart Pro, v7.3.7, ADInstruments, Bella Vista, Australia) in conscious unrestrained rats (*Protocols I* and *II*). After baseline recordings, rats from *Protocol II* were submitted to bilateral bicuculline administration into the PVN and AP and HR responses were acquired for 30 min.

Time series (~5–6 min) of resting systolic AP (SAP) and pulse interval (PI) were used to analyze pressure and HR variabilities, indicative of autonomic control of vessels and heart. The analysis in the frequency domain was made by power spectral analysis using an open‐access software (CardioSeries v2.4, (Dias 2014)). Briefly, stationary original time series were converted to equally spaced series and divided into half‐overlapping sequential sets of 512 data points (Welch periodogram). The spectrum of each segment was calculated using a fast Fourier transform (FFT) algorithm for discrete time series. The obtained spectra were integrated in low‐ frequency (LF: 0.20–0.75 Hz) and high‐frequency (HF: 0.75–3.0 Hz) domains. Previous studies analyzing LF and HF spectra in the presence/absence of sympathetic and vagal blockade showed that LF‐PAS is an index of sympathetic activity to vessels, LF‐PI indicates sympathetic + some parasympathetic modulation of the heart while HF‐PI reflects the parasympathetic modulation of the heart (Dias 2014). A recent spectral analysis study in conscious rats also showed a great agreement between pressure and heart rate variabilities performed through arterial pulse tracing and EKG time series, therefore validating this technique in laboratory animals (Dias 2014)). Results were expressed in absolute values (mmHg^2^, ms^2^) or normalized units (nu). Spontaneous baroreflex sensitivity was calculated in the time domain using the sequence method. Ramps (four or more consecutive beats) showing increase or decrease in AP (≥1 mmHg) along with increase or decrease in PI (≥1 msec) were selected for the analyses. Spontaneous baroreflex sensitivity was determined by the mean linear regression coefficient between SAP and PI values of each baroreflex sequence.

### Tissue sampling for qPCR and immunofluorescence

At the end of hemodynamic recordings, rats were deeply anesthetized (ketamine 300 mg/kg + xylazine 60 mg/kg, *i.p*) for brain tissue harvesting immediately after the cardiopulmonary arrest. Rats allocated to the qPCR study (*Protocol I*) were submitted to 5‐min transcardiac perfusion with sterile buffered saline (0.01 mol/L PB, ±30 mL/min, Peristaltic pump, Vernon Hills, IL). Brains were removed and quickly transferred to a dry‐ice box. A coronal slice of 800–1000 *μ*m taken at the hypothalamic level was immediately frozen for bilateral punching of the PVN (Cavalleri et al. 2011). Samples were collected in Trizol (Invitrogen, Life Technologies, Grand Island) and stored at −80°C for posterior processing.

Rats allocated to immunofluorescence assays (*Protocol II*) were submitted to transcardiac perfusion with Dulbecco's Modified Eagle's Medium (~300 mL of DMEM, D‐8900, Sigma‐Aldrich, St Louis MO) followed by 300 mL of 4% paraformaldehyde in 0.1 mol/L PBS, as described before (Santos et al. 2018). Briefly, brains were removed, postfixed cryoprotected and stored at 4°C until processing.

### Real‐time quantitative PCR

Temporal changes in glutamic acid decarboxylase isoforms (GAD65 and GAD67) mRNA expression within the PVN during T and S protocols were estimated by qPCR according to the technique previously described (Cavalleri et al. 2011). Briefly, 2 *μ*g/reaction of pure mRNA extract (whose integrity was verified by agarose gel electrophoresis) was used for first‐strand cDNA synthesis using SuperScript II + RNaseOUT. cDNA samples, stored (−20°C) until processing, were subjected to real‐time PCR amplification (Corbett Research System, Corbett Life Sciences, Sydney, Australia) using Platinum SYBR Green and specific nucleotides for GAD65 (sense primer: GGCAGACCAACCGCAAAATC; antisense primer: CAATCTGCTGCTAATCCAACCAT) and GAD67 (sense primer: GGTCAAATAAAGATGGTGATGGGA; antisense primer: AGGACCAGTTTGGGCACAGC). Hypoxanthine‐guanine phosphoribosyltransferase (HPRT, sense primer: TTTGCTGACCTGCTGGATTAC; antisense primer: ACTTTTAGTTCCCCCGTTGA) continually expressed in all cells and not changed by hypertension or training was used as the reporter gene (Cavalleri et al. 2011). The mRNA expression data, expressed as fold increases, were calculated by the cycle threshold (Ct) using the ∆∆Ct method (Livak and Schmittgen 2001; Pfaffl 2001). To compare genes’ expression at different times, mRNA values were normalized by the GAD65 value of the WKY group at week 0 (internal calibrator).

### Immunofluorescence assay

Sequential hypothalamic coronal sections (30 *μ*m, 1.5–2.4 mm caudal to the Bregma) were cut with a cryostat (Leica CM1850, Nussloch, Germany) and every third slice was collected in tissue culture wells with 0.01 mol/L PBS. Free‐floating sections were processed as described before (Cavalleri et al. 2011). Briefly, after a 30‐min incubation with 0.3% Triton and 10% normal Donkey serum in Tris‐PBS (Millipore Temecula, California), sections were incubated overnight with the primary antibody (rabbit anti‐GAD65 polyclonal antibody, 1:500 dilution, AB5082, Millipore). The specificity of GAD65 antibody was validated through Western Blot analysis by the manufacturer. Sections were washed in Tris‐PBS and incubated with the secondary antibody (Cy3 goat anti‐rabbit, 1:400 dilution, Jackson Immunoresearch Laboratories, Baltimore MD) for 2 h. Sections were washed again (Tris‐PBS), placed in slides and mounted with the coverslip and Slowfade^®^ Gold Antifade reagent (Life Technologies Carlsbad, CA). Negative controls omitted the primary or the secondary antibody. PVN sections were carefully examined (Leica DMLB, Wetzlar, Germany) to localize the medial and posterior PVN. Areas of interest (AOIs) of fixed size were drawn within the ventromedial and posterior PVN nuclei and acquired with identical settings. Background intensity was calculated from random adjacent areas in the neuropil and the threshold was set to pass intensities 1.5× above background immunofluorescence. The density of thresholded signal within each AOI was quantified (Image ProPlus software, Media Cybernetics, Silver spring MD) as previously described (Cavalleri et al. 2011). GAD65 immunoreactivity, expressed as integrated density (including the percent area occupied by the signal and its intensity) was obtained in 8–9 slices/rat (left and right side); values were averaged to give a mean PVN value for each rat in each experimental condition.

### Statistical analysis

Results are presented as means ± SEM. ANOVA with repeated measurements (time) were used to analyze the treadmill performance in sedentary and trained SHR and WKY groups. The effects of exercise training on hemodynamic responses and gene expression data of both groups during the 8 experimental weeks were compared by factorial ANOVA. The analysis of power spectral components in sedentary and trained SHR and WKY at the end of protocols was also made by two‐way factorial ANOVA while the effect of PVN bicuculline administration in the four groups of rats was analyzed by the paired *t* test. Fisher's LSD was the post hoc test. Correlation analyses were performed by the Pearson's statistics. All analyses were conducted using the STATISTICA software 12.0 (Vince Stat Software Inc. Palo Alto CA). Differences were considered significant at *P* < 0.05.

## Results

### Effectiveness of the training protocol

SHR exhibited a better aerobic performance than age‐matched WKY since the beginning of protocols (Table 1). Exercise training increased the aerobic capacity in both groups, with significant improvement being observed at the fourth and eighth experimental weeks. At the end of protocols, SHR‐T and WKY‐T groups exhibited a similar performance gain (+0.79 ± 0.11 and +0.74 ± 0.09 km/h, Table 1). The performance gain was decreased in the SHR‐S group (−0.35 ± 0.10 km/h), but unchanged in WKY rats kept sedentary. Since there were no major changes in sedentary groups and due to the limitation in the number of rats, the effects of sedentary protocol were only analyzed at the beginning and end of experimental weeks.

**Table 1:** Absolute values of velocities attained during maximal exercise tests on the treadmill in sedentary (S) and trained (T) normotensive (WKY) and hypertensive (SHR) rats

	WKY‐S	WKY‐T	SHR‐S	SHR‐T
Performance during S and T protocols				
Week 0 (km/h)	1.10 ± 0.0 (*n* = 20)	1.10 ± 0.03 (*n* = 55)	1.50 ± 0.05# (*n* = 20)	1.50 ± 0.03# (*n* = 55)
Week 4 (km/h)	1.10 ± 0.05 (*n* = 10)	1.50 ± 0.04*† (*n* = 30)	1.20 ± 0.07* (*n* = 10)	2.10 ± 0.07#*† (*n* = 29)
Week 8 (km/h)	1.00 ± 0.07 (*n* = 10)	1.80 ± 0.07*† (*n* = 17)	1.10 ± 0.08* (*n* = 10)	2.30 ± 0.09#*† (*n* = 16)
Gain (km/h)	−0.10 ± 0.06	+0.74 ± 0.09δ†	−0.35 ± 0.10δ	+0.79 ± 0.11δ†

Values are means ± SEM. The gain represents the difference between experimental weeks 8 and 0. The number of rats is indicated in parenthesis. Training effect: group *F*_5,270_ = 52.34, *P* < 0.001; condition *F*_5,270_ = 57.03, *P* < 0.001, interaction *F*_5,270_ = 2.91, *P* = 0.014. Significances (*P* < 0.05) are: * versus week 0, ^#^ versus WKY, ^†^ versus respective S control; ^δ^ indicates a significant change in performance gain.

### Simultaneous time‐course changes of excitatory and inhibitory gene expression within the PVN and hemodynamic parameters

**Figure 2:**
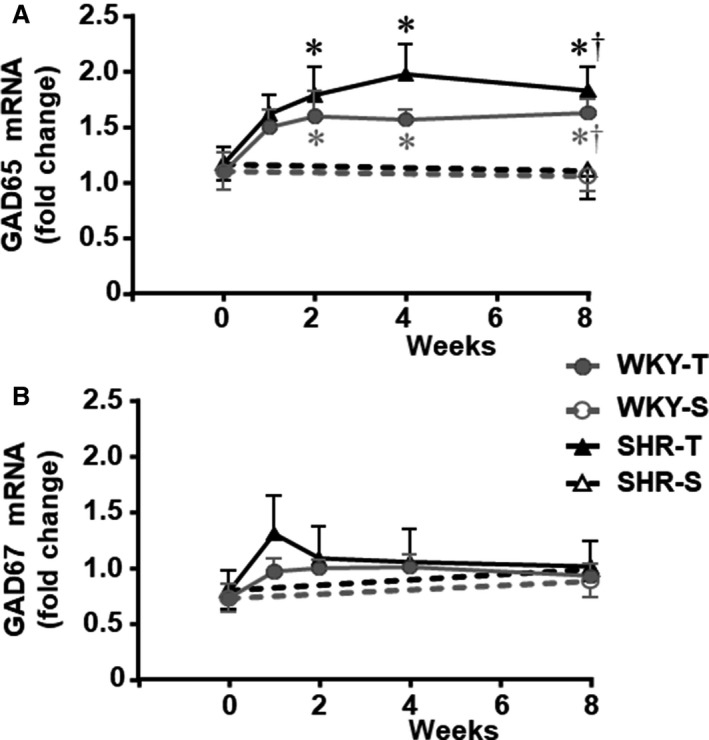
Temporal changes on GAD65 (panel A) and GAD67 (panel B) gene expression within the PVN of spontaneously hypertensive (SHR) and normotensive rats (WKY) submitted to exercise training (T) or sedentary (S) protocols for 8 weeks. Values are means of 6–8 rats/subgroup. GAD65 – *training effect:* group *F*_4,69_ = 3.64, *P* = 0.060; weeks *F*_4,69_ = 4.77, *P* = 0.002; group × week *F*_4,69_ = 0.30, *P* = 0.879; *groups comparison:* group *F*_1,53_ = 3.21, *P* = 0.079; condition *F*_1,53_ = 5.43, *P* = 0.023; weeks *F*_1,53_ = 9.04, *P* = 0.004; group × condition *F*_1,53_ = 0.01, *P* = 0.977; group × week *F*_1,53_ = 0.05, *P* = 0.825; condition × week *F*_1,53_ = 5.43, *P* = 0.024; group × condition × week *F*_1,53_ = 0.01, *P* = 0.977. GAD67 – *training effect:* group *F*_4,69_ = 1.12 *P* = 0.294; weeks *F*_4,69_ = 1.03, *P* = 0.397; group × week *F*_4,69_ = 0.18, *P* = 0.948; *groups comparison:* group *F*_1,55_ = 0.65, *P* = 0.425; condition *F*_1,55_ = 0.03, *P* = 0.856; weeks *F*_1,55_ = 2.99, *P* = 0.089; group × condition *F*_1,55_ = 0.01, *P* = 0.971; group × week *F*_1,55_ = 0.02, *P* = 0.897; condition × week *F*_1,55_ = 0.03, *P* = 0.856; group × condition × week *F*_1,55_ = 0.01, *P* = 0.971. Significance (*P* < 0.05) * versus respective week 0; † versus respective S control.

The temporal effects of T and S protocols on the main inhibitory PVN pathways in SHR and WKY groups are shown in Figure 2. Time‐course changes of GAD65 and GAD67 gene expression were analyzed at the first, second, fourth, and eighth experimental weeks. At the beginning of the experiments, GAD65 and GAD67 mRNA expression were similar in SHR‐S and WKY‐S and no changes were observed in rats kept sedentary for 8 weeks (Fig. 2A–B). In both groups, exercise training was accompanied by increased GAD65 mRNA expression into the PVN, which attained significant levels since the second up to the eighth week (SHR‐T = 1.8 ± 0.3, 2.0 ± 0.3, and 1.8 ± 0.2 fold change; WKY‐T = 1.6 ± 0.2, 1.6 ± 0.1, and 1.6 ± 0.1 fold change at weeks 2, 4, and 8 versus week 0, respectively, Fig. 2A). During the exercise protocol SHR‐T and WKY‐T exhibited only mild transient, not significant, changes in GAD67 mRNA expression within the PVN (Fig. 2B).

**Figure 3:**
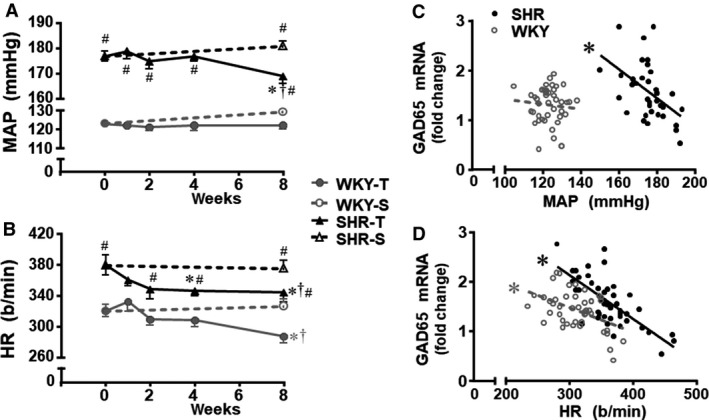
Temporal changes on hemodynamic parameters (panels A and B) and correlations between resting mean arterial pressure (MAP, panel C) and heart rate (HR, panel D) with GAD65 gene expression within the PVN of spontaneously hypertensive (SHR) and normotensive rats (WKY) submitted to exercise training (T) or sedentary (S) protocols for 8 weeks. *n* = 6–8 rats/subgroup. MAP – *training effect:* group *F*_4,98_ = 1170.43, *P* < 0.001; week *F*_4,98_ = 1.86, *P* = 0.165; group × week *F*_4,98_ = 3.31, *P* = 0,045; *groups comparison:* group *F*_1,61_ = 996.64, *P* < 0.001; condition *F*_1,61_ = 1.67, *P* = 0.201; weeks *F*_1,61_ = 0.62, *P* = 0.433; group × condition *F*_1,61_ = 3.61, *P* = 0.050; group × week *F*_1,61_ = 0.12, *P* = 0.728; condition × week *F*_1,61_ = 4.19, *P* = 0.044; group × condition × week *F*_1,61_ = 2.86, *P* = 0.096. HR – *training effect:* group *F*_4,98_ = 36.47, *P* < 0.001; week *F*_4,98_ = 3.10, *P* = 0.019; group × week *F*_4,98_ = 0.68, *P* = 0,605; *groups comparison:* group *F*_1,61_ = 50.35, *P* < 0.001; condition *F*_1,61_ = 0.51, *P* = 0.476; weeks *F*_1,61_ = 7.16, *P* = 0.009; group × condition *F*_1,61_ = 2.98, *P* = 0.049; group × week *F*_1,61_ = 0.34, *P* = 0.564; condition × week *F*_1,61_ = 2.60, *P* = 0.052; group × condition × week *F*_1,61_ = 0.89, *P* = 0.349. Significances (*P* < 0.05) are * versus respective week 0, # versus WKY, † versus respective S control. Regression equations, correlation coefficients and *P* values for GAD65 × MAP **(**panel C) are: *Y*_SHR_ = −0.029*x* + 6.71, *r* ^2^ = 0.245, *P* = 0.002; *Y*_WKY_ = −0.005*x* + 1.92, *r* ^2^ = 0.007, *P* = 0.575. Regression equations, correlation coefficients and *P* values for GAD65 × HR (panel D) are: *Y*_SHR_ = −0.009*x* + 4.78, *r* ^2^ = 0.520, *P* < 0.001; *Y*_WKY_ = −0.005*x* + 2.97, *r* ^2^ = 0.219, *P* = 0.001. In panels C and D * means a significant correlation.

In these rats, we also analyzed the time‐course changes on baseline values of MAP and HR induced by training and sedentary protocols. As expected, SHR‐S exhibited high MAP and HR levels (177 ± 2 mmHg, 379 ± 13 b/min vs. 123 ± 1 mmHg, 320 ± 8 b/min in the WKY‐S, Fig. 3A andB) at the beginning of experiments. No MAP and HR changes were observed in rats kept sedentary. In contrast, SHR‐T showed an early and progressive reduction in baseline HR, with significant decrease being observed from the second up to the eighth experimental week (on average 9% vs. week 0, Fig. 3B), coinciding with the significant increase observed in PVN GAD65 expression. In these rats the establishment of resting bradycardia preceded the partial MAP fall significant only after 8 weeks of exercise training (−6%, Fig. 3A). A progressive but late HR decrease also occurred in WKY‐T, which attained significance at the eighth experimental week (−10%, vs. respective value at week 0, Fig. 3B). Exercise training did not change MAP in the WKY group (Fig. 3A). Importantly, training‐induced HR and MAP reductions in the SHR and HR decrease in the WKY rats were negatively correlated with training‐induced augmentation in GAD65 gene expression within the PVN. As observed in Figure 3D, increased PVN GAD65 gene expression in trained SHR was correlated (*r* ^2^ = 0.520, *P* < 0.001) with the establishment of exercise‐induced resting bradycardia. Similar but weaker correlation was observed for GAD65 expression and HR reduction in the WKY group (*r* ^2^ = 0.219, *P* = 0.001). Training‐induced GAD65 mRNA augmentation into the PVN also correlated with MAP fall in SHR rats (*r* ^2^ = 0.245, *P* = 0.002, Fig. 3C).

### Changes in GABAergic tonus within the PVN: effects of hypertension and exercise training

**Figure 4:**
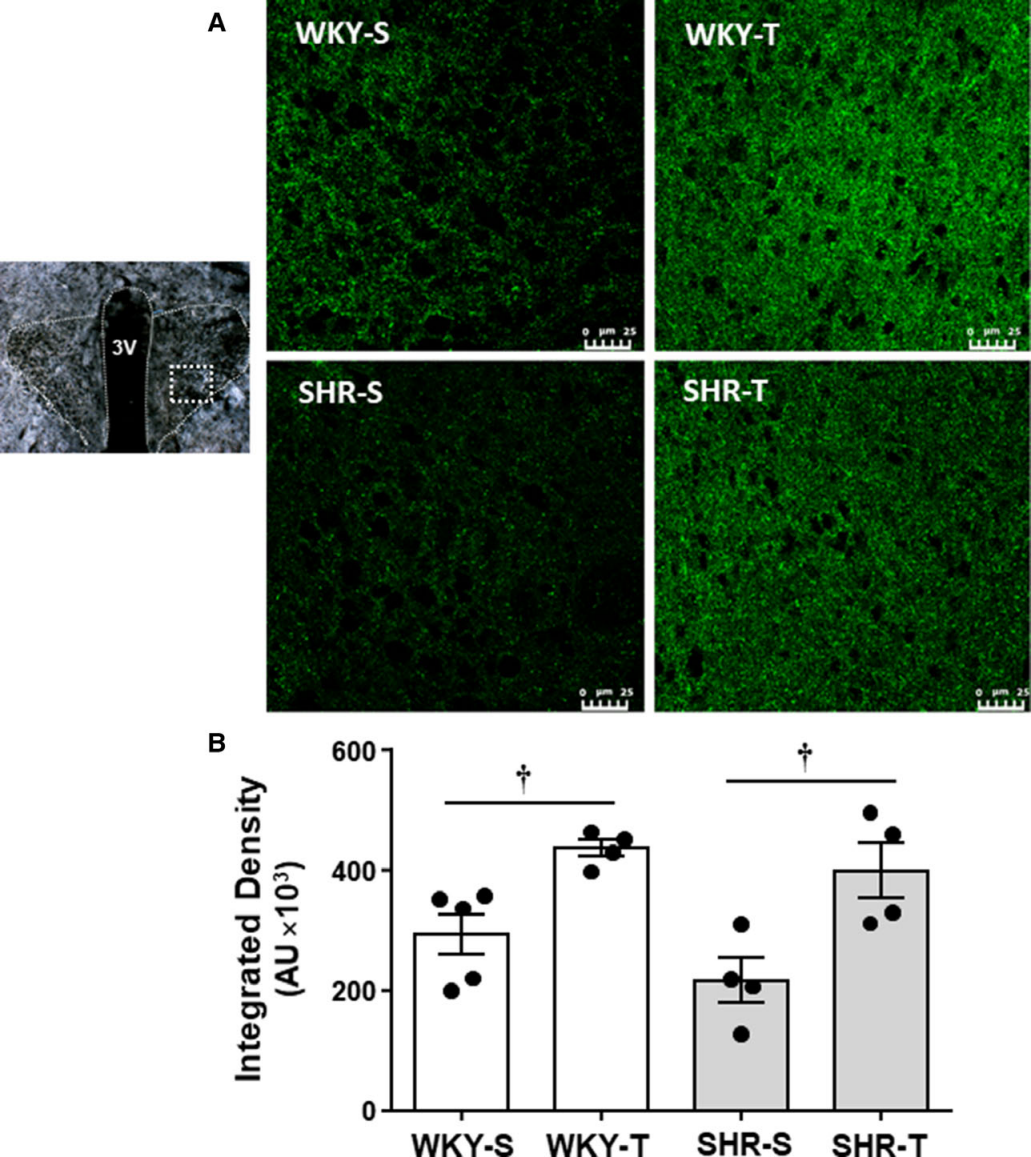
(A) Photomicrographs comparing the effects of hypertension and exercise training in GAD65 immunoreactivity (*ir*) within the PVN of spontaneously hypertensive (SHR) and normotensive rats (WKY) submitted to exercise training (T) or sedentary (S) protocols for 4 weeks. In the presence of Triton, a very dense labeling of GAD65‐immunoreactive terminals is observed in trained groups. Scale bar = 25 mm. (B) Quantification of GAD65 density within the PVN in the 4 groups of rats. Values are means of 8–9 slices/rat, 4–5 rats/group. GAD65 *ir* – *groups comparison:* group *F*_1,13_ = 2.63, *P* = 0.129; condition *F*_1,13_ = 21.43, *P* < 0.001; group × condition *F*_1,13_ = 0.33, *P* = 0.574. Significance (*P* < 0.05): † versus respective S control.

To confirm training‐induced changes in gene expression and its relationship with functional parameters, next we analyzed GAD65 protein expression within the PVN. SHR and WKY were trained or kept sedentary for 4 weeks (*Protocol II*), the exact time point in which we observed the maximal increase in GAD65 gene expression. After recording the functional parameters, half of the rats of each group were deeply anesthetized for brain harvesting. PVN was fixed, sliced and processed for GAD65 immunofluorescence. Figure 4A shows that the fluorescent signal was weaker in SHR‐S when compared to WKY‐S, but markedly increased by training in both groups. Quantitative measurements confirmed this observation showing a 32% decrease in GAD65 integrated density in the SHR‐S versus WKY‐S (*P* > 0.05, Fig. 4B) and that exercise training was accompanied by marked increases in GAD65 fluorescence in both SHR‐T and WKY‐T (+95% and +43%, respectively, when compared with respective sedentary controls, *P* < 0.05, Fig. 4B).

**Figure 5:**
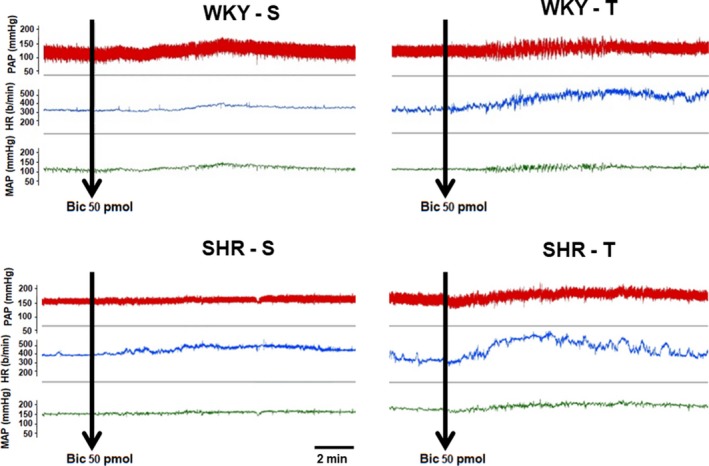
Raw tracings of pulsatile (PAP) and mean arterial pressure (MAP) and heart rate (HR) in sedentary (S) and trained (T) conscious normotensive (WKY) and spontaneously hypertensive rats (SHR) before and after bilateral bicuculline microinjection into the PVN (arrow).

**Figure 6:**
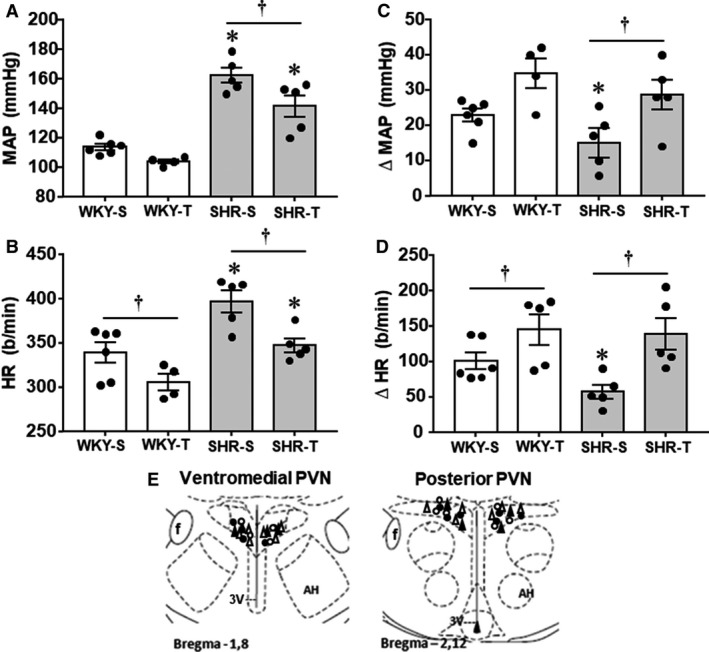
(A–D) Comparison of baseline values of mean arterial pressure (MAP, panel A) and heart rate (HR, panel B) and MAP (panel C) and HR (panel D) responses to bilateral bicuculline administration into the PVN of SHR and WKY submitted to training (T) or sedentary (S) protocol for 4 weeks. *n* = 5–6 rats/group MAP – *groups comparison*: group *F*_1,16_ = 80.54, *P* < 0.001; condition *F*_1,16_ = 10.19, *P* = 0.006; group × condition *F*_1,16_ = 1.33, *P* = 0.265. HR – *groups comparison*: group *F*_1,16_ = 20.71, *P* < 0.001, condition *F*_1,16_ = 14.14, *P* = 0.002; group × condition *F*_1,16_ = 0.58, *P* = 0.456. ∆MAP – *groups comparison*: group *F*_1,16_ = 4.37, *P* = 0.053; condition *F*_1,16_ = 14.55, *P* = 0.002; group × condition *F*_1,16_ = 0.06, *P* = 0.805. ∆HR – *groups comparison*: group *F*_1,16_ = 0.58, *P* = 0.459, condition *F*_1,16_ = 12.87, *P* = 0.003; group × condition *F*_1,16_ = 4.58, *P* = 0.049. Significances (*P* < 0.05) are * versus WKY, † versus respective S control. (E) Localization of the positive bilateral microinjections of bicuculline within the ventromedial and posterior PVN nuclei. ∆ represents the WKY and ○ the SHR rats; white symbols correspond to the sedentary and dark ones to the trained rats. Schematic diagrams are based on the Rat Brain Atlas of Paxinos and Watson (1998). F, fornix; 3V, third ventricle.

In the other half of rats of SHR‐S, SHR‐T, WKY‐S, and WKY‐T groups we investigated the functional effects endogenous GABA within the PVN on cardiovascular control. In addition, knowing that impaired GABAergic control of presympathetic PVN neurons in spontaneous hypertension is caused by an imbalance between GABA_A_ (attenuated) and GABA_B_ (enhanced) receptor function (Li and Pan 2006, 2007), we analyzed whether exercise training was able to rescue the compromised GABA_A_ receptor function in the chronic phase of hypertension. Mild and transitory (lasting approximately 30 sec) behavioral effects were observed immediately after bicuculline administration in all groups of rats. Figure 5 illustrates for one rat of each group the arterial pressure and heart rate recordings before and after bilateral bicuculline administration into the PVN. In trained groups, baseline MAP and HR (recorded in conscious rats for 30–40 min before the bicuculline treatment) showed changes equivalent to that observed in our time‐course study: SHR‐T and WKY‐T exhibited resting bradycardia (−12% and −9% reduction in basal HR, respectively, vs. respective sedentary controls, *P* < 0.05, Fig. 6B), which was accompanied by a partial MAP reduction in the SHR‐T (−13%, *P* < 0.05, Fig. 6A). Bilateral administration of bicuculline (50 pmol/100 nL) directed to the posterior and ventromedial PVN nuclei, two important preautonomic areas, caused in the four groups transient MAP and HR increases that peaked at 5–6 min and returned to baseline values 20–30 min later. Comparison of maximal HR responses (Fig. 6D) showed a significant reduction in SHR‐S (−47% vs. WKY‐S), but marked increases in HR responses in both groups submitted to exercise training (SHR‐T = +140 ± 18 b/min, WKY‐T = +147 ± 20 b/min, corresponding to increases of 169% and 48% vs. respective controls, Fig. 6D). Observe that the depressed HR response in the SHR‐S was completely rescued in the SHR‐T. MAP responses following bicuculline administration into the PVN showed a similar pattern: a smaller MAP response was observed in the SHR‐S (−36% vs. WKY‐S, *P* < 0.05, Fig. 6C), whereas larger responses were displayed by trained rats (SHR‐*T* = +29 ± 5 mmHg, WKY‐T = +35 ± 5 mmHg, corresponding to 99% and 54% increases, vs. respective S controls). Positive microinjections sites were depicted in Figure 6E.

**Figure 7:**
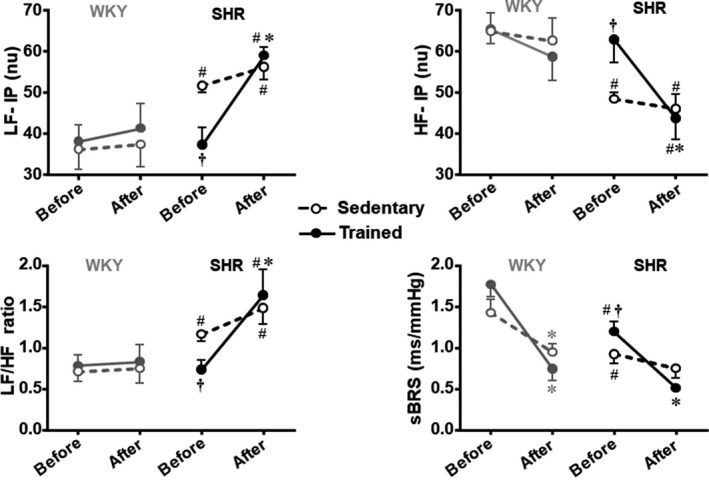
Changes in autonomic control of the heart induced by bilateral bicuculline administration into the PVN of spontaneously hypertensive (SHR) and normotensive rats (WKY) submitted to exercise training (T) or sedentary (S) protocols for 4 weeks. Graphs represent the changes in the sympathetic activity (LF‐PI), parasympathetic activity (HF‐PI) in normalized units (nu), ratio between both (LF/HF), and the spontaneous baroreflex sensitivity (sBRS) before and after bicuculline administration. *n* = 5–6 rats/group. Statistical values for groups’ comparison and bicuculline effect are shown in Table 2. Significances (*P* < 0.05) are * versus before, # versus respective WKY, † versus respective S control.

To further evaluate the relationship between PVN GABA availability and cardiovascular control, we also analyzed the autonomic responses before and after PVN bicuculline administration in the four groups of rats. Power spectral analysis before GABA_A_ receptor blockade (Table 2) revealed that sympathetic vasomotor component was significantly elevated in SHR‐S versus WKY‐S (2.7‐fold increase in LF‐SAP), with a trend to elevate the SAP variability (+42%, *P* > 0.05). Sympathetic modulation of heart was also increased in SHR‐S versus WKY‐S whereas parasympathetic activity was reduced (+43% in LF‐PI, ‐26% in HF‐PI, respectively, *P* < 0.05), therefore causing significant increase in the LF/HF ratio (+65%) with a marked decrease in spontaneous baroreflex sensitivity (−35%) and mild changes in PI variability (−19%, *P* > 0.05, Table 2). Before bicuculline administration into the PVN, exercise training significantly changed the autonomic control only in the SHR (vs. respective sedentary control): LF‐SAP and SAP variability were reduced by 55% and 53%, respectively, while LF‐PI, HF‐PI, and LF/HF ratio returned to control levels, therefore normalizing the spontaneous baroreflex sensitivity (Table 2). On the other hand, in both SHR‐T and SHR‐S GABA_A_ receptor blockade was accompanied by significant increases in sympathetic vasomotor activity and pressure variability that were larger in the trained group (LF‐SAP = +4.8‐ and +1.7‐fold, SAP variability = +3.1‐ and +1.6‐fold, respectively, Table 2). As depicted in Table 2 and compared in Figure 7, GABA_A_ receptor blockade caused robust changes in the autonomic control of the heart only in the SHR‐T group: the LF/HF ratio was markedly increased (+2.3‐fold, due to significant augmentation of LF‐PI and a large reduction in HF‐PI) with a great decrease in the spontaneous baroreflex sensitivity (0.51 ± 0.01 msec/mmHg, corresponding to a 58% decrease). In the WKY group, GABA_A_ receptor blockade also caused significant augmentation of sympathetic vasomotor activity and SAP variability (increases in 4.5‐ and 2.7‐fold for WKY‐T and WKY‐Sm, respectively, Table 2) and a significant reduction of spontaneous baroreflex sensitivity with mild, not significant, changes in LF‐PI and HF‐PI (Table 2, Fig. 7).

**Table 2:** Systolic arterial pressure (SAP) and pulse interval (PI) variabilities and respective spectral components in sedentary (S) and trained (T) normotensive (WKY) and hypertensive (SHR) rats before and after bilateral administration of bicuculline into the PVN

	PI variability (ms^2^)	LF‐PI (nu)	HF‐PI (nu)	LF/HF ratio	SAP variability (mmHg^2^)	LF‐SAP (mmHg^2^)	sBRS (ms/mmHg)
WKY‐S							
Before	75.9 ± 18.5	36.2 ± 4.9	64.9 ± 4.3	0.71 ± 0.12	26.6 ± 5.9	3.4 ± 0.7	1.42 ± 0.16
After	89.1 ± 27.1	37.4 ± 5.5	62.6 ± 5.5	0.75 ± 0.18	56.4 ± 7.6*	11.3 ± 2.6*	0.95 ± 0.10*
WKY‐T							
Before	82.3 ± 18.7	38.6 ± 3.9	65.3 ± 3.6	0.78 ± 0.13	19.2 ± 4.2	2.8 ± 0.5	1.77 ± 0.15
After	81.8 ± 39.6	41.3 ± 5.2	58.7 ± 5.9	0.83 ± 0.20	52.0 ± 20.3*	12.7 ± 3.5*	0.75 ± 0.15*
SHR‐S							
Before	61.3 ± 12.1	51.7 ± 1.6#	48.3 ± 1.6#	1.17 ± 0.09#	37.8 ± 4.2	9.1 ± 0.9#	0.92 ± 0.12#
After	85.8 ± 38.3	56.2 ± 3.2#	46.0 ± 3.4#	1.48 ± 0.20#	59.1 ± 13.5	15.5 ± 2.2*	0.75 ± 0.12
SHR‐T							
Before	90.9 ± 35.8	37.2 ± 4.2†	62.8 ± 5.6†	0.73 ± 0.11†	17.8 ± 4.2†	4.1 ± 0.4†	1.20 ± 0.12#†
After	62.6 ± 10.8	59.0 ± 2.1*#	43.7 ± 5.2*	1.64 ± 0.31*#	55.0 ± 4.5*	19.6 ± 1.3*	0.51 ± 0.01*#

Values are means ± SEM. *n* = 5–6 rats/group. Groups’ comparison for PI‐Variability: group *F*_1,36_ = 0.77, *P* = 0.517; condition *F*_1,36_ = 0.24, *P* = 0.627, group × condition *F*_1,36_ = 0.19, *P* = 0.905. LF‐PI: group *F*_1,36_ = 7.69, *P* < 0.001, condition *F*_1,36_ = 7.39, *P* = 0.010, group × condition *F*_1,36_ = 2.82, *P* = 0.052. HF‐PI: group *F*_1,36_ = 5.72, *P* = 0.003; condition *F*_1,36_ = 5.48, *P* = 0.025; group × condition *F*_1,36_ = 1.49, *P* = 0.232. LF/HF: group *F*_1,36_ = 4.77, *P* = 0.007; condition *F*_1,36_ = 6.15, *P* = 0.018; group × condition *F*_1,36_ = 2.35, *P* = 0.089. SAP‐Variability: group *F*_1,36_ = 1.29, *P* = 0.292; condition *F*_1,36_ = 19.52, *P* < 0.001; group × condition *F*_1,36_ = 0.30, *P* = 0.824. LF‐SAP: group *F*_1,36_ = 3.38, *P* = 0.028; condition *F*_1,36_ = 53.61, *P* < 0.001; group × condition *F*_1,36_ = 2.52, *P* = 0.074. sBRS: group *F*_1,36_ = 6.34, *P* = 0.001; condition *F*_1,36_ = 46.40, *P* < 0.001; group × condition *F*_1,36_ = 4.29, *P* = 0.011. Significances (*P* < 0.05) are: ^#^ versus respective WKY, ^†^ versus respective S group. * indicated a significant difference from before bicuculline administration (paired *t* test).

## Discussion

The present set of data confirmed both the depressed GABAergic modulation within autonomic areas of the sedentary SHR and its improvement by aerobic training. In addition, our data revealed that training‐induced inhibition of sympathetic activity in SHR was accompanied by the restoration of GABA_A_ receptor function within the PVN. New observations were as follow: (1) exercise training induces in both strains a sequential augmentation of GAD65 mRNA expression into the PVN that peaked at the 2nd–4th week of training, when its protein expression is largely increased, (2) training effect is specific for GAD65 expression without changing GAD67 expression, (3) training‐induced augmentation of GAD65 transcripts correlates with resting HR reduction in WKY and SHR (stronger effect) and with MAP fall (SHR only), (4) enhanced PVN GABA availability in trained groups is accompanied by decreased SAP variability, reduced sympathetic vasomotor activity and increased spontaneous baroreflex sensitivity, which are blocked by local bicuculline administration, (5) the reduced sympathetic and improved parasympathetic control of the heart observed only in the trained SHR occurred simultaneously with restored GABA_A_ receptor function in the presence of increased PVN GABA availability, (6) training‐induced recovery of ionotropic GABA receptors’ function within the PVN contributes to the improvement of autonomic control since bicuculline induces marked autonomic dysfunction.

Previous studies have already shown that sympathoexcitation in hypertension is accompanied by reduced GABAergic drive with an important imbalance between excitatory/inhibitory inputs to PVN presympathetic neurons (Kramer et al. 2001; Chen and Toney 2003; Chen et al. 2003; Jia et al. 2014; Dampney et al. 2018). Besides the loss of GABAergic neurons, a marked attenuation of GABA_A_ with simultaneous augmentation of GABA_B_ receptor function accounted for the reduced GABAergic drive into the PVN of the SHR (Li and Pan 2006; Li et al. 2008; Zhang and Mifflin 2010b). In the sedentary SHR, Li and colleagues (Li et al. 2008) have already shown that the metabotropic GABA_B_ receptor expressed presynaptically attenuated GABAergic synaptic inputs and augmented synaptic glutamate release, thus enhancing the activity of PVN presympathetic neurons and the sympathetic outflow to heart and vessels. Enhanced GABA_B_ but unchanged GABA_A_ receptor expression was observed within the nucleus of the solitary tract (NTS, another important brain autonomic area) in different models of hypertension (Zhang et al. 2007, 2009; Zhang and Mifflin 2010a,b). Whether GABA_A_ receptor depression is specific for PVN and/or the SHR model, it remains to be determined.

It is also known that training is able to change the balance between excitatory (decreased glutamatergic activity) and inhibitory (augmented GABAergic activity) inputs within the PVN thus contributing to the reduction of sympathetic hyperactivity in spontaneous and renal hypertension (Jia et al. 2014; Zhang et al. 2016). Data of the present study showed that exercise training caused a prompt and significant increase in GAD65, but did not significantly change GAD67 gene expression. It is known that GAD65 and GAD67 synthesize GABA at different locations in the neuron, at different developmental times, and for functionally different purposes (Kaufman et al. 1991; Soghomonian and Martin 1998). GAD67, spread throughout the cell, is activated early in the development and synthesizes GABA for synaptogenesis and neuronal protection (the so called metabolic pool); in contrast, GAD65, localized in nerve terminals and synapses (the vesicular pool), is activated late in life and synthesizes GABA for neurotransmission, being activated by synaptic inputs (Kaufman et al. 1991; Soghomonian and Martin 1998). Altogether these data reinforce the differential localization of GAD65 and GAD67 and suggest that repetitive sessions of exercise, by increasing PVN neuronal excitability, specifically augmented GAD65 gene and protein expression and increased GABA availability in nerve terminals, which is a strong inhibitory input for presympathetic neurons. In the present study we did not record the effects of exercise on PVN neuronal excitability, but we did before using a similar exercise protocol (Jackson et al. 2005): whole cell patch clamp recordings of preautonomic neurons (identified by retrograde labeling) showed that repetitive sessions of exercise increased intrinsic excitability and the neuronal input/output function.

Our data also revealed that training‐induced upregulation of GAD65 expression (and the consequent GABA availability at synapses) occurred simultaneously with decreased sympathetic vasomotor activity, increased spontaneous baroreflex sensitivity and reduced the resting HR, in both SHR‐T and WKY‐T. However, reduction of sympathetic and improvement of parasympathetic modulation of the heart was only observed in SHR‐T. These findings suggested that beneficial effects of training were mediated not only by increased GABA availability (observed in both SHR‐T and WKY‐T) but also by the training‐induced restoration of the depressed PVN GABA_A_ receptor function exhibited by SHR‐S, since bicuculline administration into the PVN of trained SHR blocked the improvement of the autonomic control of the heart. Indeed, in normotensive rats Hsu and collaborators (Hsu et al. 2011) demonstrated that treadmill running upregulated PVN GABAergic system by increasing GAD mRNA, augmenting both the percentage of GABAergic neurons and the gephyrin expression (a cluster protein of GABA receptors), but did not change the protein expression of GABA_A_ receptor. Our data together with these observations indicated that improvement of ionotropic receptor function is a specific response of the SHR to aerobic training and that training‐induced recovery of GABA_A_ receptor function enhances postsynaptic inhibition of presympathetic PVN neurons involved in the autonomic control of the heart. It is worth noting that GABA_A_ receptors within presympathetic PVN neurons, in addition to mediate conventional quantal synaptic transmission (IPSCs, phasic inhibition), also underlie a slower persistent tonic inhibition (likely due to extrasynaptic receptors), both currents being blocked by bicuculline (Park et al. 2007). Therefore, by recovering GABA_A_ receptor function, training augments baroreflex sensitivity and reduces sympathetic outflow to heart and vessels contributing to the appearance of resting bradycardia (since the 2nd–4th week) and a partial pressure fall (a late response) in the trained SHR. The late fall in blood pressure is conditioned not only by the reduced sympathetic activity to vessels but also by a known training‐induced outward eutrophic remodeling of arteries/arterioles (Melo et al. 2003; Amaral & Michelini 2011), both contributing to a more gradual reduction in total peripheral resistance. The reduced sympathetic activity associated to increased local release of nitric oxide (due to hyperkinetic blood flow during daily exercise sessions) is important factors determining the vascular remodeling.

It should be noted that training‐induced restoration of GABA_A_ receptor function and improvement in GABAergic modulation is just one of the neural/humoral factors conditioning sympathetic output which, in association with other factors, controls blood pressure, and heart rate levels. Although the present set of data provided functional evidence of exercise‐induced restoration of depressed GABA_A_ receptor function, we did not identify the underlying mechanism that remains to be studied.

In this study there were some caveats to be taken into consideration. We did not investigate whether exercise training alters or not GABA_B_ receptor function. To our knowledge, there is no information on the modulatory effects of aerobic training upon the expression/activity of GABA_B_ receptors within autonomic areas of hypertensive individuals. In a recent paper, Zhang and collaborators (Zhang et al. 2018) showed that electroacupuncture attenuated the increased GABA_B_ receptor expression within the NTS, reduced blood pressure, and improved baroreflex function in renal hypertensive rats. Although it is possible that reduced GABA_B_ expression/activity could contribute to training‐induced effects on PVN GABAergic neurotransmission, this effect remains to be determined. Other limitation of our study is that we did not identify the PVN presympathetic neurons by retrograde labeling. For this reason, care was taken to analyze GAD‐positive terminals and to microinject bicuculline into known autonomic areas as the ventromedial and posterior PVN nuclei, where the cell bodies of presympathetic neurons are located (Swanson & Sawchenko. 1983; Coote et al. 1998; Allen et al. 2002). Notwithstanding increased GAD65 immunoreactivity in trained rats and reduced GABAergic activity after bicuculline administration in these nuclei were accompanied by reduced and augmented sympathetic activity, respectively. Another caveat is the absence of excitatory inputs’ measurement after exercise training. The degree GABAergic release of sympathetic activity induced by bicuculline depends on the glutamatergic tone, which was not evaluated.

In conclusion, our data showed that exercise training induces in both normotensive and hypertensive rats an early and maintained increase in the expression/activity of GABAergic input to presympathetic PVN neurons. Training‐induced improvement is specific for GABA vesicular release, without noticeable changes in the metabolic pool. The increased synaptic release of GABA is accompanied by reduced sympathetic activity, improved autonomic control of heart and vessels, therefore contributing to the establishment of resting bradycardia (SHR‐T and WKY‐T) and partial pressure fall (SHR‐T). In addition, training specifically augments the inhibitory input in hypertensive rats by restoring GABA_A_ receptor function within the PVN. Training‐induced recovery of the attenuated ionotropic receptor function in SHR is an important adaptive mechanism that strengthens GABAergic modulation of sympathetic outflow in hypertension.

## Conflict of Interest

The authors declare no potential conflict of interest concerning to the research, authorship and/or publication of this article.
